# Seasonal Changes of Fish Assemblages in a Subtropical Lagoon in the SE Gulf of California

**DOI:** 10.1155/2014/968902

**Published:** 2014-01-27

**Authors:** F. Amezcua, F. Amezcua-Linares

**Affiliations:** ^1^Instituto de Ciencias del Mar y Limnología, Universidad Nacional Autónoma de México, Joel Montes Camarena S/N, Mazatlán, Sin., 82040, Mexico; ^2^Instituto de Ciencias del Mar y Limnología, Universidad Nacional Autónoma de México, Circuito Exterior S/N, Ciudad Universitaria, 04510, DF, Mexico

## Abstract

The composition and seasonal changes of the fish assemblage in a coastal lagoon system in southeastern Gulf of California were assessed from December 2001 to July 2005. A total of 20,877 organisms belonging to 191 species and 47 families were analyzed. We determined that almost all the species inhabiting the system were found; however some rare species were not captured in our study. The majority of the species found were demersal but in every season at least one pelagic or benthopelagic species showed high abundances. The moonfish, *Selene peruviana*, was the most abundant species, whilst the puffer, *Sphoeroides annulatus*, was the main species in terms of biomass. The species composition changed seasonally; results from the Simpson diversity index and the cumulative species curve show that seasonally almost all the species in the system for a given season were found. These changes were also reflected in the multivariate results. The seasonal variations could be attributed to the migration of species out of the system as they grow and the arrival of new ones, which could also be related to temperature patterns since this environmental factor changes considerably through the year.

## 1. Introduction

Estuarine areas and coastal lagoons are highly productive ecosystems which are considered to be nursery areas for many invertebrate and fish species [1–4]. Coastal lagoons are also known to serve as complimentary ecosystems in the life cycle of some species because they are used as refuges for reproducing adults. In addition, the availability of food in coastal lagoons favors the development of larvae and the growth of juvenile fish [5, 6].

Several authors have emphasized the importance of estuaries for marine fisheries. A large part of fish landings around the world consists of species that spend at least part of their lives in estuarine waters [7–9]. Species that regularly enter the lagoon to spawn or feed and those that complete their entire life cycle in the area can be considered to be dependent on lagoon systems [10].

Lagoon systems directly support essential fisheries, with the consequence that, at present, relatively few remain unexploited [11]. Estuarine ecosystems face increased stress due to fishing activities and many estuarine habitats are being destroyed rapidly [12]. In order to understand and protect these critical habitats, it is important to document the communities they support and understand the factors that naturally influence the distribution and abundance of associated species. These environments may undergo extreme fluctuations, leading to high variability in the number and abundance of fish species [13–15].

Studies undertaken in estuaries in the temperate regions of both the northern and southern hemispheres have demonstrated cyclic, seasonal changes in fish species composition as a result of seasonal changes, as well as the migration of species which use these systems as nursery habitats, into deeper water as they increase in size [1, 16–19]. Similar studies in tropical and subtropical estuarine habitats in south Florida, USA [20], México [21–23], Costa Rica [24], and Solomon Islands [25] have described fish assemblage distribution and structure in relation to seasonal variations in species number, biomass, and density and have discussed their importance as nursery areas. However, these studies fail to explain the reason of the observed changes.

Although many factors influence patterns of species composition and diversity, it has proven difficult to determine which of these covariables are most important in creating and maintaining structure within communities [26]. With the aim of addressing this question, our study describes the structure of the fish assemblage in the subtropical coastal lagoon of Santa María la Reforma (SE Gulf of California), which is one of the most important fishing grounds in the region, and its seasonal changes in relation to environmental variables. Environmental data was recorded during the surveys, which provides an opportunity to study fish assemblage patterns of diversity and abundance in relation to a suite of oceanographic variables. Specifically, water temperature, salinity, and time (months, and years) are examined in order to determine how they influence fish composition and diversity in the study system.

## 2. Material and Methods

**Figure 1:**
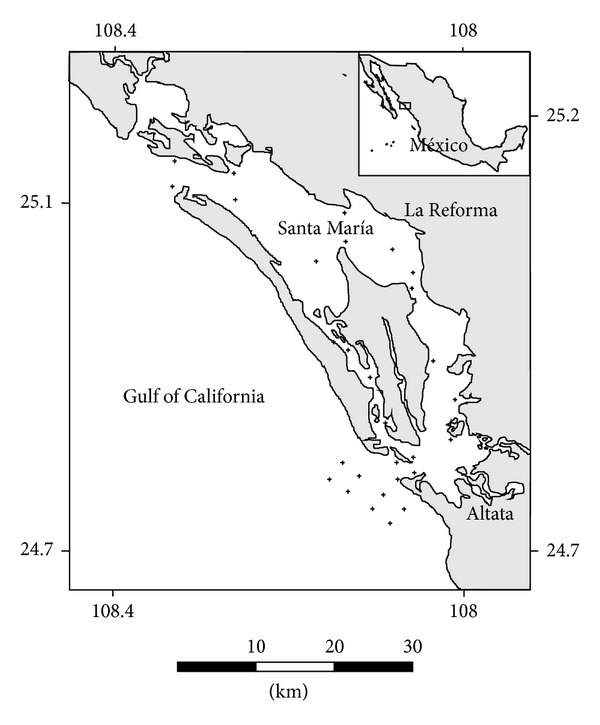
Studied area and sampling stations (dots).

Sampling was done at the coastal lagoon of Santa María la Reforma (25°04′30^”^ N-108°03′30^”^ W) on the continental shelf of the central Mexican Pacific. The National Fisheries Institute undertook biological surveys in this lagoon at 29 stations distributed in all the different environments of the system (channels, mouth, shore, and mangroves) (Figure 1). These surveys were conducted monthly from December 2001 to May 2002, in which all stations were sampled for five consecutive days during morning hours, and seasonally from 2004 to 2005, in which all stations were sampled for one day during morning hours as well.

Because the system covers a large area, daily sampling of all stations required the use of ten 7.5 m boats fitted with 115 hp outboard engines. Each boat was equipped with the three types of fishing gear commonly used in the system to catch shrimp: a shrimp trawl net with a 24 m footrope and a 50 mm liner at the codend which was used at all the stations, a 300 m long gill net fitted with a 75 mm liner which was used at all the stations, and a suripera net which is a cast net modified for trawling which was used only at the stations located in the mouth and the lagoon. A description of this fishing gear can be found in Amezcua et al. [28]. All fishing operations lasted 20 minutes and were undertaken one immediately after the other at each station before moving to the next one.

To make all tows comparable, the catch of each gear was transformed into catch-per-unit area (CPUA) estimated by dividing the total fish catch in each fishing operation by the area swept by the gear. The units were recorded as kg/ha.

To estimate the swept area, the width of each gear was recorded and then multiplied by the distance each gear was towed, estimated with the aid of a Global Positioning System (GPS). The latitude and longitude at the start and end of every fishing operation were recorded and the distance towed was estimated in nautical miles by using the equation developed by Sparre and Venema [29]:

(1) D=60((Lats−Late)2   +(Lons−Lone)2cos⁡2(0.5(Lats+Late)))1/2,

where *D* is the distance, Lat_*s*_ is the latitude at the start, Lat_*e*_ is the latitude at the end, Lon_*s*_ is the longitude at the start, and Lon_*e*_ is the longitude at the end.

This procedure was repeated for each tow with each gear, resulting in 870 × 3 distance records.

To standardize the different fishing gears, the area swept by every gear was derived from the cubic function of the geometry of each gear. The associated error between the sums of these areas was solved under the assumption of resolving all the possible areas of each gear to be able to integer them separately, so the area swept with each gear in each fishing operation was known. Then, the mean area swept by each gear type and its standard error were calculated using bootstrap estimates of the data and obtaining bootstrap samples which were assumed to approximate the distribution of values that would have arisen from repeatedly sampling the original sampled population. Each of these bootstrapped samples was treated as an independent random sample from the original population [30]. Two thousand independent bootstrap samples were generated. The bootstrap replicate of the parameter *θ*_*b*_ for each of the *b* bootstrap samples was calculated. The mean of the bootstrap replicates was calculated with the formula

(2) $$\begin{array}{c} {\overset{-}{\theta }}_{b}=\frac{\sum \theta_{b}}{b}. \end{array}$$

The standard error se_*θ*_ of the parameter was estimated as

(3) $$\begin{array}{c} {\text{se}}_{\theta }=\sqrt{\frac{\sum {\left(\theta_{b}-{\overset{-}{\theta }}_{b}\right)}^{2}}{b-1}},\;\left[\text{29}\right]. \end{array}$$

At each station, the temperature and salinity were recorded with a YSI multisensor sonde, and the fish caught by each gear were stored in plastic bags (labeled with date, station number, and the fishing gear used) and frozen. In the laboratory, fish were identified to the species level, and the total length (TL) and weight were recorded for every specimen.

**Figure 2:**
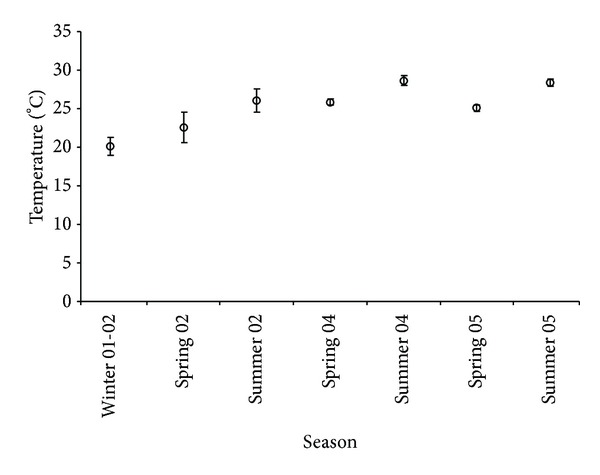
Mean temperature values (°C) during the sampled seasons and years.

Recorded temperatures were averaged seasonally and plotted into a graph to examine seasonal trends (Figure 2). The total number and biomass of fish were standardized by dividing the total fish catch in every tow by the CPUA; thus the biomass and abundance of fish per hectare were calculated. This standardized number was used for all further analyses.

The relative abundance and biomass were estimated seasonally for every species in relation to the total captured abundance and biomass, respectively [23]. Additionally, the percent occurrence of each species, defined as the proportion of months in which the species *j* was caught, was calculated using the formula

(4) $$\begin{array}{c} O_{i}=\frac{\text{no}.\;\text{of}\;\text{months}\;\text{with}\;\text{species}\;j\;}{\text{total}\;\text{no}.\;\text{of}\;\text{months}}\times 100. \end{array}$$

A randomized cumulative species curve was constructed to determine if the number of species found in the study was close to the total number of species expected in our samples [31]. The order in which samples were analyzed was randomized 1,000 times. For each new cumulative species sample, the negative exponential model proposed for species accumulation of rare plants Magurran [32] was adjusted by minimizing the negative-logarithmic likelihood via the equation

(5) $$\begin{array}{c} S_{t}=\beta_{0}\left(1-e^{-\beta_{1}t_{i}}\right), \end{array}$$

where *S*_*t*_ is the species richness at time *t*_*i*_, *β*_0_ is the asymptotic value of species richness (*S*_max⁡_) as *t* → +*∞*, and *β*_1_ is the rate at which the maximum value is attained. For both parameters, the bias corrected percentile 95% confidence interval was calculated [30, 33].

To describe the monthly species-abundance relationship, the observed data was fitted to a species-abundance model. Although species-abundance data can be described with different distributions [34], this relationship is usually examined using the following four models: (a) geometric series, (b) log series, (c) log normal, and (d) broken stick. Further details of these models can be found in Magurran [32]. To determine the goodness of fit, a Chi-Squared test of the observed and expected observations was performed. If *P* < 0.05, then the distributions were significantly different at the 5% level indicating that our data did not fit that model [35]. To graphically observe the relationship, the frequency of species was plotted in relation to abundance.

Diversity was estimated using the Simpson's index (*D*) which gives the probability that any two individuals draw at random from an infinitely large community belong to the same species. The form of the index appropriate for a finite community is

(6) $$\begin{array}{c} D=\sum \left(\frac{n_{i}\left[n_{i}-1\right]}{N\left[N-1\right]}\right), \end{array}$$

where *n*_*i*_ is the number of individuals in the *i*th species and *N* is the total number of individuals. As *D* increases diversity decreases; therefore this index is usually expressed as −ln⁡(*D*) following Rosenzweig [36] who explains that this transformation is easily interpretable, reflects the underlying diversity, and is independent of sample size. This index also captures the variance of the species-abundance distribution [32]. The confidence intervals for the Simpson's index were generated using a bootstrap procedure, which is a technique that allows the estimation of sample variability by resampling from the empirical probability distribution defined by a single sample. The bias corrected 95% confidence interval was obtained from 1000 bootstrap samples of species [30, 33].

The fish assemblage composition was compared among the environmental factors of season (winter was defined as the period from December to February, spring from March to May, and summer from June to July; no data was available from August to November) and year using the ordination method of multidimensional scaling analysis (MDS) on Bray Curtis similarity coefficients calculated from 4th-root-transformed-abundance data. Because every season had a mean temperature, the temperature factor was included into the season factor. To test for differences in the faunal composition between the factors, an analysis of similarity (ANOSIM) was employed using the *R*-statistic values for pairwise comparisons to determine the degree of dissimilarity between groups [37]. Similarity of percentages (SIMPER) was used to determine which species account for most of the dissimilarities between the compositions in the different seasons and years when they were significantly different [38, 39]. MDS was performed by Statistica 6.0 [40] from a similarity matrix obtained from PRIMER; ANOSIM and SIMPER analyses were performed by the PRIMER suite of programs [39].

## 3. Results

In total, 20,877 organisms belonging to 47 families and 191 species were analyzed (Table 1). In terms of abundance, the five most important species were *Selene peruviana*, which accounted for 23.08% of the total abundance, followed by *Eucinostomus entomelas* (7.74%), *Etropus crossotus* (3.92%), *Diapterus peruvianus* (3.72%), and *Eucinostomus gracilis* (3.67%). In terms of biomass, the five most important species were *Sphoeroides annulatus*, which accounted for 10.47% of the total biomass, followed by *E. entomelas* (8.92%), *Rhinobatos glaucostigma* (5.11%), *Urotrygon chilensis* (5.04%), and *S. peruviana *(3.77%).

The abundance and biomass of the species changed seasonally (Table 2). During winter 2001-2002 and spring 2002 the most abundant species was *E. entomelas*; however its abundance was much higher during winter than spring. During spring and summer 2004 the five most abundant species were the same, with similar relative abundances and a high abundance of *S. peruviana, *which accounted for approximately 50% of the total abundance in both seasons. In spring 2005, the most abundant species was *Anchoa walkeri, *and in summer 2005 it was *Pomadasys nitidus. *The most abundant species changed between 2001 and 2002 samples and 2004 and 2005 samples. While *E. entomelas *and *D. peruvianus* were characteristic of the first two seasons, the species *E. gracilis*, *E. crossotus* and *P. nitidus* were more characteristic of the years 2004 and 2005.

The biomass changed seasonally; during winter 2001-2002 and spring 2002 the species *E. entomelas* and *Menticirrhus elongates *were amongst the five species with the highest biomass. These results were similar to abundance results for the same time periods. *S. annulatus *was among the top five species in terms of biomass in all seasons of the study except for both summer seasons analyzed which were characterized by *P. nitidus* and *D. peruvianus. S. peruviana*, which was the most abundant species in both analyzed seasons of 2004, was the species with the highest biomass during spring 2004 but was not amongst the top five species with higher biomasses during the summer of the same year. *Stellifer fuerthii* increased its biomass from spring to summer 2004, at which point it became the species with the highest relative biomass. *E. crossotus*, which was highly abundant during the seasons of 2004-2005, had a high biomass only during summer 2005. In general the most abundant species also showed the highest biomasses. The species *Achirus mazatlanus, D. peruvianus, E. crossotus, E. entomelas, Larimus effulgens, Pliosteostoma lutipinnis, P. nitidus, Pseudupeneus grandisquamis,* and *S. peruviana* were captured in all the sampling months; therefore their percentage of occurrence was 100%.

The majority of the most abundant species, both in terms of biomass and number, were demersal organisms. A notable exception is the genus *Selene* spp., a benthopelagic species that showed high abundance in almost all the sampled seasons with the exception of spring 2002 and 2005, when it was not amongst the most abundant species. However, in these two seasons the pelagic species *Pliosteostoma lutipinnis* and *Anchovia macrolepidota *during spring 2002 and *A. walkeri* during spring 2005 showed high abundances.

Temperature and salinity data were available from all the seasons sampled as well as for summer 2002. Temperature varied seasonally, with lower temperatures occurring during winter than summer (mean values of 20°C and 29°C, resp.) (Figure 2). The temperature was higher during spring and summer 2004 and 2005 than during the same seasons in 2002. Salinity was fairly uniform, ranging from 35.1 to 35.4 in all seasons and years sampled; therefore no further analyses were conducted using this factor.

**Figure 3:**
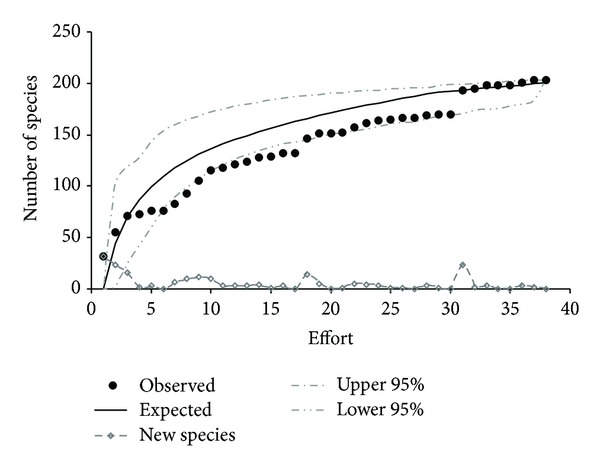
Species accumulation curve, number of new species, and expected number of species according to the von Holdridge richness model.

The expected number of species was 200 according to the von Holdridge richness model, which is higher than the observed number of species (191). An asymptote was not reached with the species accumulation curve, indicating that some rare species were not collected (Figure 3). It is also possible to observe that throughout the study, three previous asymptotes were reached: February 2002, March 2002, and May 2004. After these months the number of new species increased.

**Figure 4:**
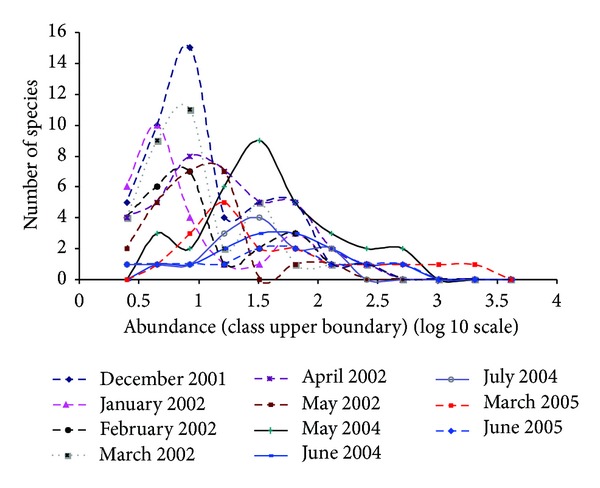
Frequency of species in relation to abundance in the different sampling months.

In all months, the fish assemblage adjusted to a log normal model, indicating that there is a mode containing the midabundant species which are the majority and a small number of rare and very abundant species which are located to the left and to the right of this mode, respectively (Table 3 and Figure 4). Since very rare species are not fully represented in a finite sample, usually the left-hand tail of the distribution is not present as is the case of the plots from 2001 to 2002. The months of 2001 and 2002 were characterized by having many species with fewer individuals, as opposed to the other years in which less species were found but with more individuals.

**Figure 5:**
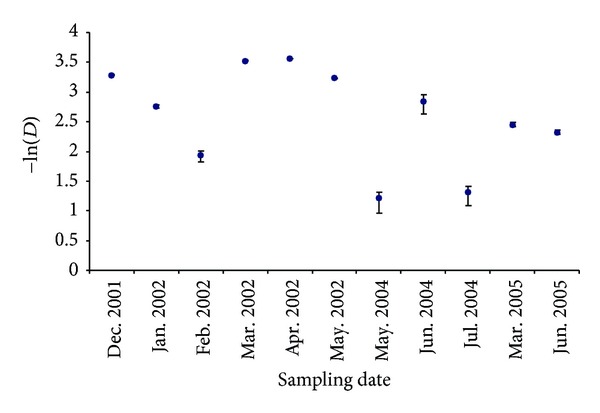
Values of the Simpson index (−ln⁡*D*) in the different sampled seasons (confidence interval 95%).

The Simpson's diversity index varied from 1.21 during May 2004 to 3.56 during April 2002. Comparisons between months in different years were in general not possible, but March and May 2002 showed higher values of diversity than during the same months in the following sampled years (Figure 5). Diversity decreased from December 2001 to February 2002 and increased during spring 2002. During 2004 diversity was generally lower with the exception of June 2004 when it was around 2.8, which is similar to spring 2002. During 2005, the diversity was 2.45 in March and 2.32 in June and showed the same trend observed during spring 2002, but with lower values.

**Figure 6:**
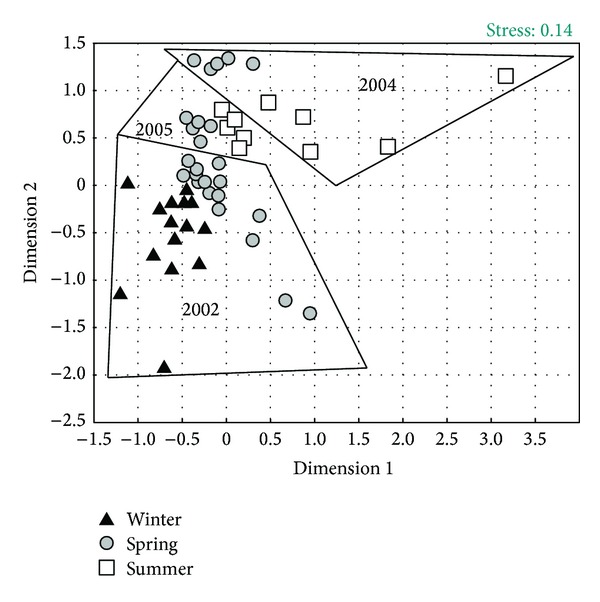
MDS plot showing the ordination of the samples according to season and year.

Season and year influenced the arrangement of the fish assemblage, and groups were formed according to these two factors (MDS plot, stress = 0.14) (Figure 6); data from the different seasons was grouped together, as well as data from the same years. These groups were corroborated by the ANOSIM; data from winter was significantly different from that of spring (*R*-statistic = 0.778, *P* < 0.1) and summer (*R*-statistic = 0.878, *P* < 0.1), with no significant differences between spring and summer seasons (*R*-statistic = 0.0, *P* > 0.5). The data from 2002 was significantly different from the data of 2004 (*R*-statistic = 0.985, *P* < 0.1) and 2005 (*R*-statistic = 0.979, *P* < 0.1), with no differences found between 2004 and 2005 (*R*-statistic = 0.002, *P* > 0.5). The species responsible for these differences varied seasonally. SIMPER results indicated that *Opisthopterus dovii* and *Peprilus medius* were more abundant during spring, and *E. currani* and *Sphoeroides lobatus* were more abundant during summer than winter. In terms of annual differences, *E. crossotus* was more abundant during 2004-2005 than during 2002, *E. gracilis* was more abundant during 2004, and *P. nitidus* was more abundant during 2005 than during 2002.

## 4. Discussion

To our knowledge, this is the most comprehensive study of the ichthyofauna in a coastal lagoon in the Gulf of California. From our results, it can be considered to be a representative description of the general composition of juveniles and adult fish inhabiting this system. Previous studies have not been undertaken in this area; therefore comparisons with previous work cannot be made. Neither is possible to determine if the total number of species has increased or decreased.

The total number of fish species in the system remains speculative but we consider the estimate of 191 species found in this study close to the total number of species that inhabited the system during the sampling period, and only rare species were not captured. But considering that the data fitted to a log normal distribution we can conclude that our sample size was adequate in all months which allow us to unveil this distribution [32].

We are confident that we captured the majority of the fish species in the system because the three types of fishing gears that we used permitted us to capture a wide range of lengths. Although the catchability is likely to vary with size within and between species, the use of these three gears, which operate in different parts of the water column and with different mesh sizes, allowed us to capture fish from 3 to 103 cm. The shrimp trawl net catches demersal and benthopelagic species from small to large, the gill net captures usually large size benthopelagic species although small individuals can also be captured, and the suripera net captures small demersal and benthopelagic fish species because of its reduced mesh size.

Amezcua et al. [28] also concluded that these three gears catch fish from the entire water column as well as individuals of a wide range of sizes. A reason we might have missed species in our sampling is that our sampling program was not continuous. We are missing information from 2003 and various seasons of the other years included in the study. We could have also increased the number of species captured by increasing the survey periods, so that diel activities were accounted for.

Studies of this kind in similar systems in the eastern Gulf of California are scarce but similar systems elsewhere in the region might present a similar diversity of species. Balart et al. [22] report 109 species in Ohuira, Topolobampo, and Santa María lagoons, which are situated north of the area of our study. In addition, Chan Gonzalez [41] reports 55 species from El Verde, Amezcua-Linares [42] reports 60 species in Huizache-Caimanero, and Alvarez-Rubio et al. [43] report 76 species in Teacapán-Agua Brava. These systems are located south of our study site. Balart et al. [22] and Chan Gonzalez [41] analyzed commercial catches to produce a list of species and did not include effort as variable. Amezcua-Linares [42] and Alvarez-Rubio et al. [43] sampled approximately 100 stations during a one-year period using three fishing gears at each station: a trawl net, a gill net, and a seine net. The fishing gears they used are similar to the ones we used in our study, but the sampling effort in our study was higher since we managed to sample more than 1500 stations during the period of our study. If the previous studies had used effort similar to that of this study, the number of species found in those studies might have been higher. Unfortunately, the previous studies did not include species accumulation curves or other analyses that would give an idea of the total number of species, so we are unable to determine if the number of species they found is close to the potential total or not.

The majority of the fish species inhabiting the system are demersal, although in most seasons pelagic or benthopelagic species also showed high abundances. The genus *Selene* spp. was highly abundant in most seasons and during the year 2004. It is known that this species is a common resident in these systems [44], but an explanation of its increased abundance during 2004 cannot be given. During spring 2002 and 2005 this species was not amongst the most abundant species, but the pelagic species *Pliosteostoma lutipinnis* and *A. macrolepidota *during 2002 and *A. walkeri* were very abundant during 2004. Castro-Aguirre et al. [44] report the entrance of these species to the estuarine system as a common behavior apparently associated with the temperature of the sea water at those times. The reason that these small pelagic species were not very abundant during spring 2004 might be the high numbers of *S. peruviana* that were already occupying the habitat and therefore precluding a high abundance of other species. This would indicate that the pelagic habitat in the estuarine habitats is a limiting factor as opposed to the demersal one, but further research is necessary to test this hypothesis.

The fish assemblage of the lagoon system of Santa Maria la Reforma showed annual and seasonal variations. This was observed in the contrasting values of diversity (*D*′) and the groups formed using multivariate analyses. The diversity changed monthly indicating changes in the species composition. These changes in diversity help explain the species accumulation curve in which the number of species increased after reaching an asymptote in certain months, indicating that the fish assemblage changed seasonally, with new species arriving the system through the year. These increases in the number of species coincide with increases in the diversity index and in the number of new species, which occurred from February to March 2002, indicating the arrival of more species that had not been recorded previously. Similar results occurred in June 2004 and March 2005.

These results indicate that the lagoon system is used by a wide variety of fish species but that the use by each species differs through the year depending on their ecology, for example, the formation of spawning aggregations or migration behavior of larger fish migrating out of the system as they grow.

The multivariate results clearly indicate a seasonal transition through the year, which seems to be related to seasonal migration patterns of the fish fauna, with a diversity that varies as some fish species leave the system, which could explain the decrease in the diversity, and others arrive it, which could explain the suddenly increase in the diversity, pointing to a differential use of this system by the different fish species; however further research is needed to corroborate this assumption, but it might be possible that this is occurring considering that previous studies have reported that these kind of systems show a high seasonal stability and adaptation of the species to variations in temperature, where seasonal patterns are maintained even during warming events [45–47].

Our results also show annual variations in the fish assemblage of the studied system, which was different from 2001-2002 to 2004-2005. The reason for the annual changes could be related to the timing of our samples, since during the years 2001-2002 most of the sampling was undertaken during the winter and spring, and during the following years most of the sampling was undertaken during summer, so when the years are compared, the differences could be a result of seasonal differences rather than annual ones.

Our study clearly shows a seasonal succession in the fish assemblage in the system and it leaves the hypothesis that these changes could be related to a partitioning of the habitat by the different species using the habitat through the year. It is necessary to consider that the temperature shows considerable fluctuations through the year, so this factor might also be important in determining the composition of the fish assemblage. Changes in abundance and species composition occur frequently in fish communities sharing neighboring biogeographical areas, as a result of migratory movements related to climate and oceanographic changes. The area of our study is a transition zone between the ichthyofauna of the Mexican province, which goes from the Gulf of Tehuantepec to Topolobampo (north of the studied area), and the Gulf of California province, that extends from Topolobampo to the north [48]. In this sense, the fish assemblage found during winter could be representatives of the Gulf of California province, whilst the fish assemblage present during summer could be representative of the Mexican province, with transitions between these seasons, but a detailed analysis of the distribution of the species present in each season is needed to corroborate this assumption.

## Figures and Tables

**Table 1:** Relative abundance (*A*%), relative biomass (*B*%), and frequency of occurrence (*O*%) of fish in Santa María la Reforma coastal lagoon, Gulf of California.

Species	*A*%	*B*%	*O*%
Class Chondrichthyes			
Order Torpediniformes			
Family Narcinidae			
*Narcine entemedor *Jordan and Starks, 1895	0.02	0.04	9.09
Order Rajiformes			
Family Rhinobatidae			
*Rhinobatos glaucostigma* Jordan and Gilbert, 1883	1.23	5.11	90.91
*Rhinobatos productus* Ayres, 1854	0.02	0.01	18.18
*Zapteryx exasperata* (Jordan and Gilbert, 1880)	0.01	0.11	18.18
Family Dasyatidae			
*Dasyatis brevis* (Garman, 1880)	0.01	0.26	9.09
*Dasyatis longa* (Garman, 1880)	0.08	0.69	54.55
Family Urolophidae			
*Urobatis halleri* (Cooper, 1863)	0.52	0.82	72.73
*Urobatis maculatus* Garman, 1913	0.01	0.02	18.18
*Urotrygon aspidura* (Jordan and Gilbert, 1882)	<0.01	0.01	9.09
*Urotrygon chilensis* (Günther, 1872)	2.5	5.04	90.91
*Urotrygon munda* Gill, 1863	0.14	0.27	54.55
*Urotrygon nana* Miyake and McEachran, 1988	1.05	2.15	81.82
*Urotrygon rogersi* (Jordan and Starks, 1895)	0.53	0.88	63.64
Family Gymnuridae			
*Gymnura marmorata* (Cooper, 1864)	0.28	1.47	63.64
Family Myliobatidae			
*Rhinoptera steindachneri* Evermann and Jenkins, 1891	<0.01	<0.01	9.09
Class Actinopterygii			
Order Albuliformes			
Family Albulidae			
*Albula nemoptera* (Fowler, 1911)	1	2.78	54.55
*Albula vulpes* (Linnaeus, 1758)	0.85	2.46	54.55
Order Anguilliformes			
Family Muraenidae			
*Gymnothorax panamensis* (Steindachner, 1876)	0.11	0.51	54.55
Family Congridae			
*Rhynchoconger nitens* (Jordan and Bollman, 1890)	0.05	0.09	27.27
Family Ophichthidae			
*Bascanichthys panamensis* Meek and Hildebrand, 1923	0.01	0.1	9.09
*Echiophis brunneus* (Castro-Aguirre and Suárez de los Cobos, 1983)	<0.01	<0.01	9.09
*Ophichthus zophochir* Jordan and Gilbert, 1882	0.02	0.04	27.27
*Pseudomyrophis micropinna* Wade, 1946	<0.01	<0.01	9.09
Order Clupeiformes			
Family Pristigasteridae			
*Pliosteostoma lutipinnis* (Jordan and Gilbert, 1882)	1.82	1.29	100.00
*Opisthopterus dovii* (Günther, 1868)	0.44	0.23	54.55
Family Engraulidae			
*Anchoa argentivittata* (Regan, 1904)	0.01	<0.01	18.18
*Anchoa helleri* (Hubbs, 1921)	0.11	0.03	36.36
*Anchoa mundeola* (Gilbert and Pierson, 1898)	0.28	0.08	36.36
*Anchoa nasus* (Kner and Steindachner, 1867)	0.37	0.11	54.55
*Anchoa walkeri* Baldwin and Chang, 1970	1.45	0.45	63.64
*Anchovia macrolepidota* (Kner, 1863)	1.17	0.57	72.73
*Cetengraulis mysticetus* (Günther, 1867)	0.08	0.03	18.18
*Engraulis mordax* Girard, 1854	0.02	<0.01	9.09
Family Clupeidae			
*Lile stolifera* (Jordan and Gilbert, 1882)	0.01	0.39	27.27
*Opisthonema libertate* (Günther, 1867)	0.23	0.31	54.55
*Opisthonema medirastre* Berry and Barrett, 1963	0.14	0.15	36.36
Order Siluriformes			
Family Ariidae			
*Ariopsis guatemalensis* (Günther, 1864)	0.01	0.03	18.18
*Ariopsis seemanni* (Günther, 1864)	0.16	0.36	18.18
*Cathorops dasycephalus* Günther, 1864	<0.01	0.01	9.09
*Occidentarius platypogon* (Günther, 1864)	0.26	0.58	54.55
*Bagre panamensis* Gill, 1863	0.28	0.43	63.64
*Cathorops liropus* (Bristol, 1896)	0.07	0.07	36.36
*Notarius troschelii* (Gill, 1863)	0.03	0.01	9.09
Order Aulopiformes			
Family Synodontidae			
*Synodus evermanni* Jordan and Bollman, 1890	<0.01	<0.01	9.09
*Synodus scituliceps* Jordan and Gilbert, 1882	0.88	1.85	90.91
Order Ophidiiformes			
Family Ophidiidae			
*Lepophidium pardale* (Gilbert, 1890)	0.02	0.03	9.09
*Lepophidium prorates* (Jordan and Bollman, 1890)	0.02	0.02	18.18
*Otophidium indefatigabile* Jordan and Bollman, 1890	<0.01	<0.01	18.18
Order Batrachoidiformes			
Family Batrachoididae			
*Porichthys analis* Hubbs and Schultz, 1939	0.55	0.65	54.55
Order Lophiiformes			
Family Lophiidae			
*Lophiodes caulinaris* (Garman, 1899)	0.1	0.04	27.27
Family Antennariidae			
*Antennarius avalonis* Jordan and Starks, 1907	0.07	0.03	18.18
Order Beloniformes			
Family Hemiramphidae			
*Hemiramphus saltator* Gilbert and Starks, 1904	<0.01	<0.01	9.09
Order Syngnathiformes			
Family Fistulariidae			
*Fistularia corneta* Gilbert and Starks, 1904	0.02	0.01	27.27
Family Syngnathidae			
*Hippocampus ingens* Girard, 1858	0.04	0.01	45.45
Order Scorpaeniformes			
Family Scorpaenidae			
*Pontinus sierra* (Gilbert, 1890)	0.1	0.03	54.55
*Scorpaena mystes* Jordan and Starks, 1895	0.1	0.05	18.18
*Scorpaena sonorae* Jenkins and Evermann, 1889	0.1	<0.01	9.09
Family Triglidae			
*Bellator loxias* (Jordan, 1897)	0.03	0.01	9.09
*Bellator xenisma* (Jordan and Bollman, 1890)	0.2	0.16	63.64
*Prionotus albirostris* Jordan and Bollman, 1890	0.01	<0.01	18.18
*Prionotus birostratus* Richardson, 1844	<0.01	0.01	9.09
*Prionotus horrens* Richardson, 1844	0.13	0.04	27.27
*Prionotus ruscarius* Gilbert and Starks, 1904	0.28	0.44	63.64
*Prionotus stephanophrys* Lockington, 1881	0.49	0.32	63.64
Order Perciformes			
Family Centropomidae			
*Centropomus nigrescens* Günther, 1864	<0.01	0.01	9.09
*Centropomus robalito* Jordan and Gilbert, 1882	0.06	0.07	45.45
Family Serranidae			
*Diplectrum eumelum* Rosenblatt and Johnson, 1974	0.22	0.37	36.36
*Diplectrum euryplectrum* Jordan and Bollman, 1890	0.08	0.12	27.27
*Diplectrum labarum* Rosenblatt and Johnson, 1974	0.01	0.01	18.18
*Diplectrum macropoma* (Günther, 1864)	0.09	0.07	18.18
*Diplectrum pacificum* Meek and Hildebrand, 1925	0.2	0.29	45.45
*Diplectrum rostrum* Bortone, 1974	0.02	0.03	9.09
*Diplectrum sciurus* Gilbert, 1892	0.01	0.01	9.09
*Epinephelus analogus * Gill, 1863	0.21	0.41	54.55
*Epinephelus exsul* (Fowler, 1944)	0.01	0.01	9.09
*Mycteroperca rosacea* (Streets, 1877)	<0.01	0.01	9.09
*Paralabrax maculatofasciatus* (Steindachner, 1868)	0.11	0.21	54.55
Family Nematistiidae			
*Nematistius pectoralis* Gill, 1862	0.19	0.59	18.18
Family Carangidae			
*Carangoides otrynter* (Jordan and Gilbert, 1883)	0.1	0.13	54.55
*Caranx caballus* Günther, 1868	0.23	0.37	45.45
*Caranx caninus* (Günther, 1867)	0.49	0.79	54.55
*Caranx vinctus* Jordan and Gilbert, 1882	0.29	0.41	63.64
*Chloroscombrus orqueta* Jordan and Gilbert, 1883	0.2	0.12	45.45
*Decapterus muroadsi * (Temminck and Schlegel, 1844)	<0.01	<0.01	9.09
*Hemicaranx leucurus* (Günther, 1864)	0.05	0.06	27.27
*Hemicaranx zelotes* Gilbert, 1898	0.03	0.05	27.27
*Oligoplites altus* (Günther, 1868)	0.46	0.91	36.36
*Oligoplites refulgens* Gilbert and Starks, 1904	0.11	0.10	63.64
*Oligoplites saurus* (Bloch and Schneider, 1801)	0.11	0.22	27.27
*Selar crumenophthalmus* (Bloch, 1793)	0.13	0.33	27.27
*Selene brevoortii* (Gill, 1863)	2.29	1.05	81.82
*Selene oerstedii* Lütken, 1880	0.02	0.01	18.18
*Selene peruviana* (Guichenot, 1866)	23.08	3.77	100.00
*Trachinotus kennedyi* Steindachner, 1876	0.07	0.16	45.45
*Trachinotus paitensis* Cuvier, 1832	0.04	0.09	27.27
Family Lutjanidae			
*Hoplopagrus guentherii* Gill, 1862	0.02	0.08	9.09
*Lutjanus argentiventris* (Peters, 1869)	0.01	0.02	18.18
*Lutjanus guttatus* (Steindachner, 1869)	0.12	0.18	45.45
*Lutjanus novemfasciatus* Gill, 1862	<0.01	<0.01	9.09
Family Gerreidae			
*Diapterus aureolus* (Jordan and Gilbert, 1882)	0.13	0.04	18.18
*Diapterus peruvianus* (Cuvier, 1830)	3.72	3.62	100.00
*Eucinostomus argenteus* Baird and Girard, 1855	1.16	1.36	63.64
*Eucinostomus currani* Zauranec, 1980	0.97	0.62	72.73
*Eucinostomus entomelas* Zauranec, 1980	7.76	8.99	100.00
*Eucinostomus gracilis* (Gill, 1862)	3.67	1.57	72.73
*Eugerres axillaris* (Günther, 1864)	0.1	0.05	9.09
*Eugerres lineatus* (Humboldt, 1821)	<0.01	<0.01	9.09
*Gerres cinereus* (Walbaum, 1792)	0.09	0.06	36.36
Family Haemulidae			
*Conodon serrifer* Jordan and Gilbert, 1882	0.04	0.06	45.45
*Haemulon scudderii * Gill, 1862	0.09	0.15	27.27
*Haemulon sexfasciatum* Gill, 1862	0.06	0.09	18.18
*Haemulopsis elongatus* (Steindachner, 1879)	0.01	0.01	9.09
*Haemulopsis leuciscus* (Günther, 1864)	0.15	0.27	45.45
*Haemulopsis nitidus* (Steindachner, 1869)	0.46	0.19	18.18
*Microlepidotus brevipinnis* (Steindachner, 1869)	0.39	0.23	27.27
*Orthopristis cantharinus* (Jenyns, 1840)	0.07	0.11	36.36
*Orthopristis chalceus* (Günther, 1864)	0.03	0.05	36.36
*Orthopristis reddingi* Jordan and Richardson, 1895	0.22	0.39	36.36
*Haemulopsis axillaris* (Steindachner, 1869)	0.07	0.04	36.36
*Pomadasys branickii* (Steindachner, 1879)	0.51	0.73	81.82
*Haemulopsis elongatus* (Steindachner, 1879)	0.27	0.35	54.55
*Haemulopsis leuciscus* (Günther, 1864)	0.36	0.81	63.64
*Pomadasys macracanthus* (Günther, 1864)	0.02	0.05	18.18
*Haemulopsis nitidus* (Steindachner, 1869)	2.86	2.51	100.00
*Pomadasys panamensis* (Steindachner, 1876)	2.77	2.80	90.91
Family Polynemidae			
*Polydactylus approximans* (Lay and Bennett, 1839)	0.51	0.39	81.82
Family Sciaenidae			
*Bairdiella icistia* (Jordan and Gilbert, 1882)	0.01	<0.01	18.18
*Corvula macrops* (Steindachner, 1876)	0.03	0.01	9.09
*Cynoscion reticulatus* (Günther, 1864)	0.27	0.62	81.82
*Cynoscion parvipinnis* Ayres, 1861	0.03	0.13	18.18
*Cynoscion stolzmanni* (Steindachner, 1879)	0.1	0.20	27.27
*Elattarchus archidium* (Jordan and Gilbert, 1882)	0.09	0.08	45.45
*Isopisthus remifer* Jordan and Gilbert, 1882	0.25	0.45	63.64
*Larimus acclivis* Jordan and Bristol, 1898	0.44	0.28	72.73
*Larimus argenteus* (Gill, 1863)	0.06	0.04	18.18
*Larimus effulgens* Gilbert, 1898	0.68	0.68	100.00
*Larimus pacificus* Jordan and Bollman, 1890	0.24	0.12	54.55
*Menticirrhus elongatus* (Günther, 1864)	0.9	3.31	54.55
*Menticirrhus nasus* (Günther, 1868)	0.2	0.55	72.73
*Menticirrhus panamensis* (Steindachner, 1876)	0.04	0.10	27.27
*Micropogonias altipinnis* (Günther, 1864)	0.13	0.09	27.27
*Ophioscion imiceps* (Jordan and Gilbert, 1882)	0.09	0.10	18.18
*Ophioscion strabo* Gilbert, 1897	0.08	0.11	45.45
*Stellifer ericymba* (Jordan and Gilbert, 1882)	0.02	0.04	27.27
*Stellifer fuerthii* (Steindachner, 1876)	2.21	1.17	63.64
*Stellifer illecebrosus* Gilbert, 1898	0.29	0.22	54.55
*Umbrina xanti* Gill, 1862	0.01	0.04	18.18
Family Mullidae			
*Mulloidichthys dentatus* (Gill, 1862)	0.06	0.05	18.18
*Pseudupeneus grandisquamis* (Gill, 1863)	1.14	0.64	100.00
Family Mugilidae			
*Mugil cephalus* Linnaeus, 1758	0.11	0.2	36.36
*Mugil curema* Valenciennes, 1836	0.11	0.28	54.55
Family Ephippidae			
*Chaetodipterus zonatus* (Girard, 1858)	1.36	1.2	90.91
*Parapsettus panamensis* (Steindachner, 1876)	<0.01	<0.01	9.09
Family Chaetodontidae			
*Chaetodon humeralis* Günther, 1860	0.11	0.02	36.36
Family Pomacanthidae			
*Pomacanthus zonipectus* (Gill, 1862)	<0.01	0.01	9.09
Family Sphyraenidae			
*Sphyraena ensis* Jordan and Gilbert, 1882	0.06	0.09	45.45
Family Uranoscopidae			
*Kathetostoma averruncus* Jordan and Bollman, 1890	0.04	0.03	18.18
Family Gobiidae			
*Bollmannia chlamydes* Jordan, 1890	<0.01	<0.01	9.09
Family Trichiuridae			
*Trichiurus nitens* Garman, 1899	0.09	0.08	9.09
Family Scombridae			
*Scomber japonicus* Houttuyn, 1782	0.05	0.28	27.27
*Scomberomorus sierra* Jordan and Starks, 1895	0.63	2.03	54.55
Family Stromateidae			
*Peprilus medius* (Peters, 1869)	0.25	0.45	63.64
*Peprilus simillimus* (Ayres, 1860)	0.01	0.01	9.09
*Peprilus snyderi* Gilbert and Starks, 1904	0.09	0.14	63.64
Order Pleuronectiformes			
Family Bothidae			
*Bothus constellatus* (Jordan, 1889)	0.09	0.08	36.36
Family Paralichthyidae			
*Ancylopsetta dendritica* Gilbert, 1890	0.01	0.01	18.18
*Citharichthys fragilis* Gilbert, 1890	<0.01	<0.01	9.09
*Citharichthys gilberti* Jenkins and Evermann, 1889	0.68	0.34	81.82
*Citharichthys platophrys* Gilbert, 1891	0.01	0.01	9.09
*Citharichthys xanthostigma* Gilbert, 1890	0.03	0.01	9.09
*Cyclopsetta panamensis* (Steindachner, 1876)	0.55	0.65	72.73
*Cyclopsetta querna* (Jordan and Bollman, 1890)	0.25	0.47	81.82
*Etropus crossotus* Jordan and Gilbert, 1882	4.30	1.80	100.00
*Hippoglossina bollmani* Gilbert, 1890	0.02	0.04	9.09
*Paralichthys woolmani* Jordan and Williams, 1897	0.02	0.06	36.36
*Syacium latifrons* (Jordan and Gilbert, 1882)	0.01	0.01	9.09
*Syacium ovale* (Günther, 1864)	2.16	1.20	81.82
Family Achiridae			
*Achirus mazatlanus* (Steindachner, 1869)	1.3	0.65	100.00
*Trinectes fonsecensis* (Günther, 1862)	0.01	<0.01	9.09
Family Cynoglossidae			
*Symphurus atramentatus* Jordan and Bollman, 1890	0.01	0.01	18.18
*Symphurus atricaudus* (Jordan and Gilbert, 1880)	0.03	0.01	9.09
*Symphurus elongatus* (Günther, 1868)	0.33	0.13	27.27
*Symphurus leei* Jordan and Bollman, 1890	0.01	0.01	9.09
*Symphurus melanurus* Clark, 1936	0.17	0.07	9.09
*Symphurus prolatinaris* Munroe, Nizinski, and Mahadeva, 1991	0.01	0.01	9.09
Order Tetraodontiformes			
Family Balistidae			
*Balistes polylepis* Steindachner, 1876	0.37	0.5	72.73
*Pseudobalistes naufragium* (Jordan and Starks, 1895)	0.02	<0.01	18.18
Family Tetraodontidae			
*Canthigaster punctatissima* (Günther, 1870)	0.01	<0.01	9.09
*Sphoeroides annulatus* (Jenyns, 1842)	3.33	10.54	81.82
*Sphoeroides lobatus* (Steindachner, 1870)	1.28	0.83	81.82

**Table 2:** Percentage of the top five species in terms of abundance and biomass in each sampled season and its known habitat (H) [27].

Abundance								
Winter 2001-2002	%	H	Spring 2002	%	H	Spring 2004	%	H

*Eucinostomus entomelas *	22	D	*Eucinostomus entomelas *	6	D	*Selene peruviana *	53	B
*Sphoeroides annulatus *	8	D	*Pliosteostoma lutipinnis *	5	P	*Eucinostomus gracilis *	7	D
*Selene brevoortii *	8	B	*Diapterus peruvianus *	5	D	*Etropus crossotus *	6	D
*Diapterus peruvianus *	6	D	*Anchovia macrolepidota *	4	P	*Stellifer fuerthii *	4	D
*Urotrygon chilensis *	4	D	*Pomadasys panamensis *	4	D	*Pomadasys nitidus *	3	D

Summer 2004	%	H	Spring 2005	%	H	Summer 2005	%	H

*Selene peruviana *	50	B	*Anchoa walkeri *	20	P	*Pomadasys nitidus *	22	D
*Eucinostomus gracilis *	7	D	*Cyclopsetta panamensis *	16	D	*Etropus crossotus *	13	D
*Etropus crossotus *	5	D	*Etropus crossotus *	7	D	*Diapterus peruvianus *	12	D
*Stellifer fuerthii *	5	D	*Symphurus melanurus *	6	D	*Selene peruviana *	9	B
*Pomadasys nitidus *	3	D	*Stellifer illecebrosus *	5	D	*Eucinostomus gracilis *	8	D

Biomass								

Winter 2001-2002	%	H	Spring 2002	%	H	Spring 2004	%	H

*Eucinostomus entomelas *	19	D	*Sphoeroides annulatus *	10	D	*Selene peruviana *	22	B
*Sphoeroides annulatus *	12	D	*Rhinobatos glaucostigma *	5	D	*Sphoeroides annulatus *	11	D
*Urotrygon chilensis *	8	D	*Eucinostomus entomelas *	5	D	*Rhinobatos glaucostigma *	7	D
*Albula nemoptera *	5	D	*Menticirrhus elongatus *	4	D	*Eucinostomus gracilis *	6	D
*Menticirrhus elongatus *	5	D	*Albula vulpes *	4	D	*Stellifer fuerthii *	5	D

Summer 2004	%	H	Spring 2005	%	H	Summer 2005	%	H

*Stellifer fuerthii *	13	D	*Sphoeroides annulatus *	16	D	*Pomadasys nitidus *	17	D
*Pomadasys nitidus *	10	D	*Cyclopsetta panamensis *	13	D	*Diapterus peruvianus *	16	D
*Diapterus peruvianus *	8	D	*Urotrygon chilensis *	7	D	*Rhinobatos glaucostigma *	6	D
*Pomadasys branickii *	6	D	*Pomadasys panamensis *	7	D	*Gymnura marmorata *	6	D
*Cynoscion stolzmanni *	5	D	*Urotrygon nana *	6	D	*Etropus crossotus *	5	D

D: demersal; B: benthopelagic; P: pelagic.

**Table 3:** Results obtained when fitting the log normal distribution to our data and results of the chi-squared tests of the observed and expected observations.

	Dec. 02	Jan. 02	Feb. 02	Mar. 02	Apr. 02	May. 02	May. 04	Jun. 04	July. 04	Mar. 05	Jun. 05
Obs. Log_10_ *M*	0.81	0.76	0.76	0.88	0.93	1.12	1.77	1.37	1.11	1.71	1.98
Obs. Log_10_ *S* ^2^	0.32	0.48	0.42	0.35	0.36	0.33	0.64	0.16	0.37	0.56	0.48
Est. Log_10_ *M*	0.73	0.44	0.57	0.8	0.86	1.08	1.73	1.37	1.05	1.69	1.98
Est. Log_10_ *S* ^2^	0.4	0.82	0.62	0.45	0.45	0.39	0.73	0.16	0.45	0.61	0.48
Total pred. sp.	67.4	106.06	92.32	111.55	111.63	81.05	104.9	31	32.7	54.3	49.03
Total obs. sp.	64	84	80	106	107	80	104	31	32	54	49
*λ* diversity	107.03	117.04	117.03	167.03	166.73	130.6	123.05	76.85	48.96	69.31	70.48
*χ* ^2^	2.59	3.94	9.13	9.8	5.9	7.95	2.15	1.78	7.62	4.3	13.22
D. F.	6	8	9	8	6	7	12	6	8	10	11
*P* value	0.86	0.86	0.43	0.28	0.43	0.34	0.71	0.94	0.47	0.93	0.28

Obs.: observed; est.: estimated; pred.: predicted; sp.: species; *M*: mean; *S* ^2^: variance; D. F.: degrees of freedom.
